# How Do Psychiatry Residents View Their Training in Spain? A Mixed-Method Survey

**DOI:** 10.62641/aep.v53i1.1760

**Published:** 2025-01-05

**Authors:** Juan Pablo Carrasco, Jon-Inaki Etxeandia-Pradera, José Esteve, Eduardo Jesús Aguilar

**Affiliations:** ^1^Psychiatry Department, Consorcio Hospitalario Provincial de Castellón, 12002 Castellón, Spain; ^2^Psychiatry Department, Hospital Clínico Universitario de Valencia, 46010 Valencia, Spain; ^3^Research Group on Psychiatry and Neurodegenerative Diseases, INCLIVA-Instituto de Investigación Sanitaria, 28009 Valencia, Spain; ^4^CIBERSAM-Centro de Investigación Biomédica en Red de Salud Mental, 28029 Madrid, Spain; ^5^Medicine Department, Faculty of Medicine, University of Valencia, 46010 Valencia, Spain

**Keywords:** training, perception, opinion, quality, resident

## Abstract

**Background:**

Efforts to improve psychiatry training must incorporate residents' assessment of their training. This study sought to collect the opinion of residents about the program that has been in force in Spain since 2008, until the current transition to a new plan.

**Methods:**

The authors conducted an online survey of psychiatry residents in Spain, asking about their formative and working conditions. Based on previous research and the national training programme, it was distributed electronically to resident representatives of the National Board of Psychiatry. This descriptive, cross-sectional study used a mixed-methods (quantitative and qualitative) approach, following standard procedures for data analysis.

**Results:**

A total of 109 residents from 67 training units responded to the survey. The average score for satisfaction with their training was 6.84 (standard deviation (SE) = 2.4; the maximum possible score was 10). Psychotherapy was considered the area with the greatest need for improvement, while the rotations that participants would most like to be extended were child psychiatry and addictions. It was reported that rotation durations established by the national programme were not fulfilled in 38.5% of cases, while the required direct supervision for first-year residents was not fulfilled in 77.1% of cases. Regarding working conditions, 47.7% of the residents reported that they exceeded the maximum working time established by European law.

**Conclusions:**

Psychiatry residents in Spain perceive certain areas of their training as deficient, especially those related to psychotherapy and clinical supervision, and they consider that their working time is excessive. The approval of the new training programme opens up an interesting opportunity for improvement.

## Introduction

Despite some differences between countries, becoming a psychiatrist usually involves two consecutive phases of medical education: undergraduate (medical school) and postgraduate (psychiatry training or residency) [1, 2]. Psychiatry training in Spain, which has been the subject of various analyses in recent years [3, 4, 5], is currently undergoing a period of transition. The previous programme—that has been in place since 2008 [6] and whose last generation of residents will complete their training in 2026—has been replaced by new and differentiated adult or child and adolescent psychiatry programmes, which have been in force since 2023 [7, 8].

### International Studies on the Perspectives of Psychiatry Residents

Institutions such as the World Federation for Medical Education acknowledge that it is essential to have the perspective of current or recent trainees in order to improve training frameworks [1]. Therefore, a series of papers have collected the views of psychiatry residents from a variety of countries (e.g., USA, Canada, UK) and have covered three main areas: (a) general aspects of training (e.g., supervision, educational environment, or ethics), highlighting both the importance of clinical supervision and residents’ perception that it is usually insufficient [9, 10, 11, 12, 13]; (b) psychotherapy training, the most frequently studied specific training area, with residents consistently reporting significant deficiencies [14, 15, 16, 17, 18]; and (c) other specific training areas (e.g., emergency psychiatry, research, psychosomatic disorders, or electroconvulsive therapy), where residents have reported both learning achievements and areas for improvement or deficient skills [19, 20, 21, 22, 23, 24, 25, 26, 27, 28].

### Spanish Studies on the Perspectives of Psychiatry Residents

In Spain, only two studies have included the perspective of psychiatry residents on their training. Providing a general perspective about formative aspects, they were published in 1998 and 2011 by the National Board of Psychiatry, which reports to the Ministry of Health [29, 30]. Since then, no new studies have been carried out to replicate, update, or expand these data.

The paucity of studies means that several aspects of psychiatry residents’ perceptions of their training have so far remained unexplored. According to the Spanish regulations on postgraduate medical training for all specialties (including psychiatry), residents must have direct supervision during their first training year, and this is gradually reduced as the resident assumes more responsibility over the following years [31]. This regulation also establishes that residents have a labour-training contract [32], but working conditions for medical residents in Spain usually breach European regulations regarding maximum working hours (48 hours per week) and the minimum rest time after being on call [33, 34]. No studies have yet been carried out regarding the supervision and working conditions of psychiatry residents in Spain.

In this context, this study aimed to capture the current opinion of psychiatry residents in Spain regarding their training, considering both the educational dimension and working conditions. It aimed to describe (quantitatively and qualitatively) the residents’ perceptions of their training in terms of competence acquisition, clinical rotations, supervision, and working conditions.

## Materials and Methods

We conducted a descriptive, cross-sectional study that consisted of a mixed-methods online survey (i.e., it had both quantitative and qualitative elements) for psychiatry residents who were undergoing training in Spain during the first quarter of 2023.

### Sample

As there are approximately 1200 psychiatry residents in Spain [35], we calculated that the minimum sample size to obtain representative results was 79 (confidence level: 95%; precision: ± 0.3 around the mean). We followed standardized sample calculations [36] and revised a standard error in a previous significant study of 1.4 [37]. In addition, for qualitative questions, we followed standard saturation procedures to estimate when sufficient responses had been obtained [38].

### Survey

A tailored questionnaire was designed specifically for this study. The survey was constructed following the standard methodology used in most previous descriptive, mixed-methods, and exploratory studies on the opinions of psychiatry residents [11, 13, 14, 15, 19, 20, 23, 24, 26, 29, 30]. Based on this previous literature, the national psychiatry training programme approved in 2008 [6], and the authors’ experience, the most relevant areas and questions were selected. As detailed in Table 1, we identified four main areas of inquiry: (1) training objectives [9, 10, 11, 12, 13, 18, 19, 20, 21, 22, 23, 24, 26, 27, 28, 29, 30], (2) rotations [24, 25, 29, 30], (3) supervision [14, 15, 16, 17, 29, 30], and (4) working conditions [33, 34]. These four selected areas corresponded to the core sections (1–11) of the official programme. Both rating scale questions (score range: 1–10) and open-ended questions were developed to better gather the views of residents regarding their training in each of these four blocks, which is in line with mixed-methods studies [38, 39, 40].

**Table 1.** **Survey**.

1. Training objectives		
	How do you rate the level of skills acquired during your residency?	10-point rating scale
	How do you rate the training of cross-cutting skills during your residency?	10-point rating scale
	Which skills could be strengthened in your training process?	Open-ended
2. Clinical rotatories		
	Do the rotation times described in the training plan adhere to in your unit?	Yes/no
	Which rotations require more time to learn the necessary objectives?	Open-ended
	Which rotations would be interesting to introduce in your training process as a psychiatrist?	Open-ended
3. Supervision		
	How do you rate the supervision and progressive acquisition of objectives during your residency?	10-point rating scale
	How often have you been supervised by an attending physician during your first year of residency?	Always/often/sometimes/never
	What do you think could be improved in this process of resident supervision and training?	Open-ended
4. Working conditions		
	How many on-call shifts do you usually do per month?	10-point rating scale
	How often do you rest during the morning immediately following an on-call shift?	Always/often/sometimes/never
	How many on-call shifts do you believe should be mandatory per month?	10-point rating scale

We developed this survey using the Google Forms platform (https://www.google.com/forms/about/). We then disseminated it electronically through the representatives of psychiatry residents of the National Board of Psychiatry between January and March 2023. The survey included an informed consent clause covering the use of the data for educational and research purposes. The ethics of this study was exempted according to the guidelines of the Ethics Committee of the University of Valencia, fully complying with the corresponding ethical standards and conducted in strict accordance with the ethical principles outlined in the Declaration of Helsinki.

### Analysis

The first and third authors compiled and assessed the quantitative and qualitative responses; the latter were analysed manually using standardised thematic analysis and information labeling procedures [38], with discrepancies being resolved through consensus among the authors. Meetings were held at the beginning, middle, and end of the analysis process, presenting the labels and themes outline of each author, contrasting differences and similarities, and receiving input from the second and fourth authors. Finally, the labels were quantified, and the corresponding tables and infographics were designed. We did not use specific software for qualitative analysis due to the small size of the qualitative text responses. SPSS v.21 IBM Corp., Armonk, NY, USA, was used for statistical analysis of both quantitative and qualitative data.

## Results

A total of 109 psychiatry residents from 67 training units responded to the survey; thus, the survey comprised approximately 10% of the psychiatry residents and 50% of the training units that provide psychiatry residency in Spain (numbering 1100 and 135, respectively). None of the training units represented more than 4% of the total sample, and 16 of the 17 autonomous communities or regions of Spain with psychiatry residents were represented; the only exception was La Rioja (four residents in total, 0.3%). The majority of the participants were from Catalonia (31.2%) and the communities of Valencia (21.1%) and Madrid (16.5%), the three regions with the most psychiatry residents overall (Table 2). As for the postgraduate year of study (PGY), most of the participants were PGY-3 (33.9%), followed by PGY-2 (25.7%), PGY-4 (22.0%), and PGY-1 (18.3%). The obtained sample size exceeded the minimum calculated to be considered representative. In addition, in the qualitative questions, saturation was reached in approximately 70 answers.

**Table 2.** **Sample**.

Region	Training unit	PR* per year	Total PR	Survey participants	Survey sample proportion (%)
Andalusia	H. De Poniente	1	4	1	0.92
Andalusia	H. Virgen De La Macarena	3	12	1	0.92
Aragon	H. Obispo Polanco	1	4	1	0.92
Aragon	H. Ntra. Sra. Del Pilar	1	4	1	0.92
Aragon	H. Royo Villanova	1	4	2	1.83
Asturias	H. Universitario De Cabueñes	2	8	1	0.92
Balearic Islands	H. Universitario Son Espases	2	8	1	0.92
Basque Country	H. Universitario Basurto	3	12	2	1.83
Basque Country	H. Universitario Donostia	3	12	2	1.83
Basque Country	H. Galdakao-Usansolo	2	8	1	0.92
Basque Country	H. Universitario De Araba	3	12	1	0.92
Canary Islands	H. Universitario De Canarias	2	8	3	2.75
Canary Islands	H. Ntra. Sra. De La Candelaria	2	8	2	1.83
Cantabria	H. Marqués De Valdecilla	3	12	1	0.92
Castile and Leon	Complejo Asist. Salamanca	3	12	2	1.83
Castile and Leon	H. El Bierzo	1	4	1	0.92
Castilla-La Mancha	Hospital Albacete	2	8	1	0.92
Castilla-La Mancha	H. General De Ciudad Real	2	8	1	0.92
Catalonia	H. Germans Trias I Pujol	2	8	1	0.92
Catalonia	H. Del Mar- Parc De Salut Mar	6	24	4	3.67
Catalonia	H. Universitari Vall D’hebron	4	16	3	2.75
Catalonia	H. Clínic De Barcelona	4	16	3	2.75
Catalonia	H. De La Santa Creu — Sant Pau	4	16	2	1.83
Catalonia	H. Universitari De Bellvitge	3	12	1	0.92
Catalonia	H. Arnau De Vilanova De Lleida	4	16	1	0.92
Catalonia	Xarxa Assistencial Manresa	2	8	1	0.92
Catalonia	Udm Salut Mental Sagrat Cor	2	8	4	3.67
Catalonia	Institut Pere Mata	4	16	3	2.75
Catalonia	Corporació Sanitaria Parc Taulí	2	8	2	1.83
Catalonia	Institut D’assistencia Sanitaria	4	16	3	2.75
Catalonia	Udm Salud Mental Benito Menni	4	16	3	2.75
Catalonia	Parc Sanitari Sant Joan De Déu	5	20	1	0.92
Catalonia	H. Universitar— Mútua Terrassa	2	8	1	0.92
Catalonia	Consorci Hospitalari De Vic	1	4	1	0.92
Extremadura	Del Área De Salud De Plasencia	4	16	1	0.92
Galicia	De Pontevedra	1	4	1	0.92
Galicia	Udm Santiago De Compostela	3	12	1	0.92
Madrid	H. Príncipe De Asturias	4	16	2	1.83
Madrid	H. Universitario Del Henares	2	8	1	0.92
Madrid	H. Universitario De Getafe	2	8	1	0.92
Madrid	H. José Germain	2	8	2	1.83
Madrid	H. Universitario De La Princesa	3	12	2	1.83
Madrid	H. Universitario Ramón Y Cajal	4	16	1	0.92
Madrid	H. Universitario La Paz Madrid	4	16	1	0.92
Madrid	H. Universitario 12 De Octubre	4	16	2	1.83
Madrid	H. Dr. Rodríguez Lafora	4	16	2	1.83
Madrid	H. Gregorio Marañón	5	20	1	0.92
Madrid	H. Clínico San Carlos	4	16	1	0.92
Madrid	H. Universitario De Fuenlabrada	2	8	1	0.92
Madrid	H. Universitario Puerta De Hierro	4	16	1	0.92
Murcia	C.H, Sta. M2 Del Rosell	1	4	1	0.92
Murcia	H. Morales Meseguer	1	4	1	0.92
Murcia	H. Virgen De La Arrixaca	2	8	2	1.83
Navarre	Clínica Universidad De Navarra	2	8	2	1.83
Valencia	H. Universitario La Ribera	1	4	1	0.92
Valencia	Hosiptal De Castellón	3	12	2	1.83
Valencia	H. Universitario De Elche	2	8	1	0.92
Valencia	H. De Elda	2	8	1	0.92
Valencia	H. De La Vega Baja	1	4	2	1.83
Valencia	H. Sant Joan De Alicante	2	8	2	1.83
Valencia	H. Arnau De Vilanova	2	8	1	0.92
Valencia	H. General De Valencia	2	8	2	1.83
Valencia	H. Universitari I Politecnic La Fe	3	12	3	2.75
Valencia	H. Clínico De Valencia	3	12	3	2.75
Valencia	H. Universitario Doctor Peset	2	8	3	2.75
Valencia	H. Marina Baixa	2	8	1	0.92
Valencia	H. Lluis Alcanyis	1	4	1	0.92
Total				109	100.00

*PR, psychiatry residents.

### Training Objectives

**Fig. 1.**
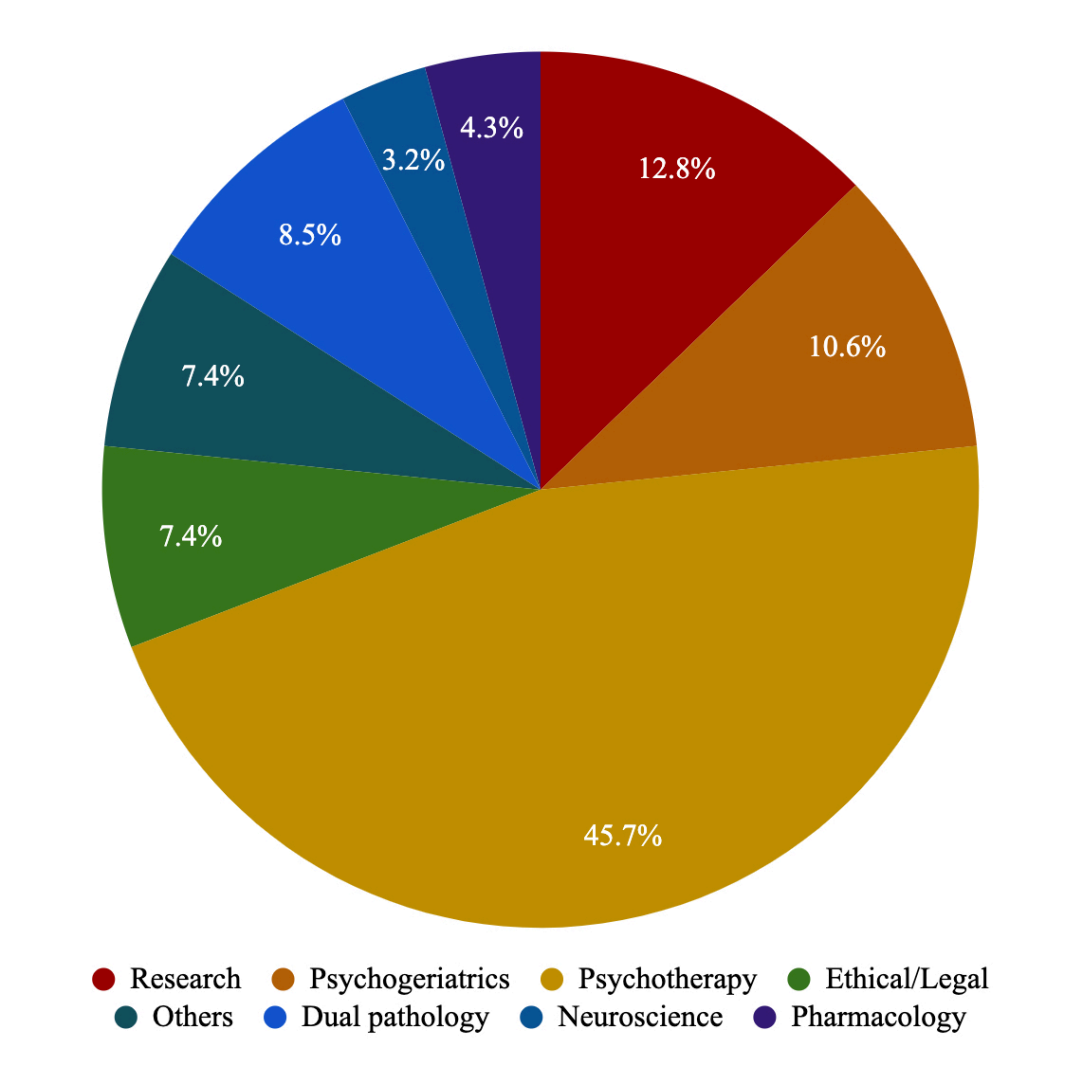
**Percentage of psychiatry residents in Spain who considered that the given competencies need to be improved**.

The residents’ average satisfaction score regarding the competencies acquired throughout their training was 6.9 ± 2.4 (mean ± standard deviation (SE)), whereas the average satisfaction regarding the promotion of transversal objectives (e.g., communication, ethics) in their training process was 5.4 ± 2.6. Fig. 1 summarises residents’ opinions on which objectives they felt are currently less developed. The competency that residents identified as by far the worst developed in their programs was psychotherapy (45.7% of residents), followed by research (12.8%), psychogeriatrics (10.6%), and dual pathology (8.5%).

### Rotations

**Fig. 2.**
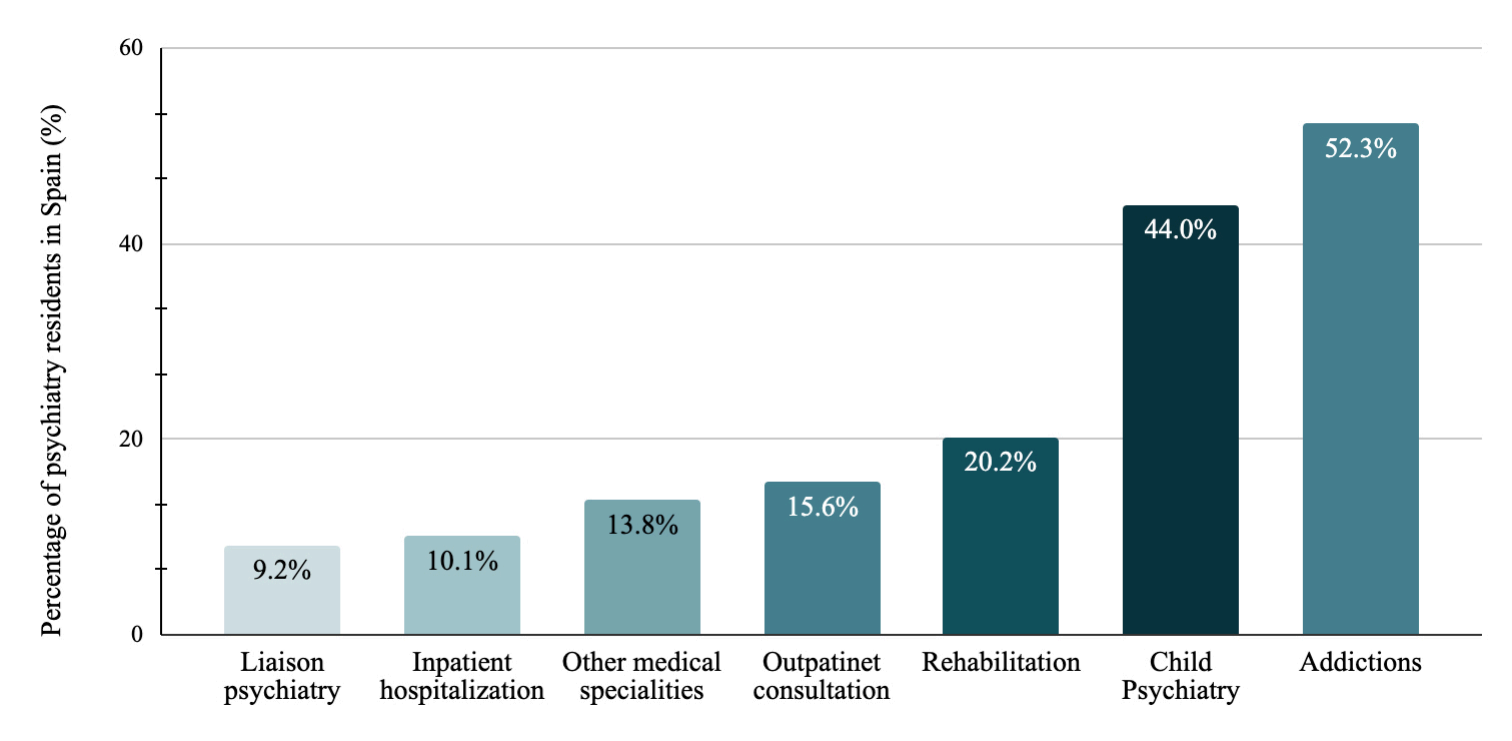
**Percentage of psychiatry residents in Spain who considered that specific rotations included in the psychiatry programme should be extended in time**.

**Fig. 3.**
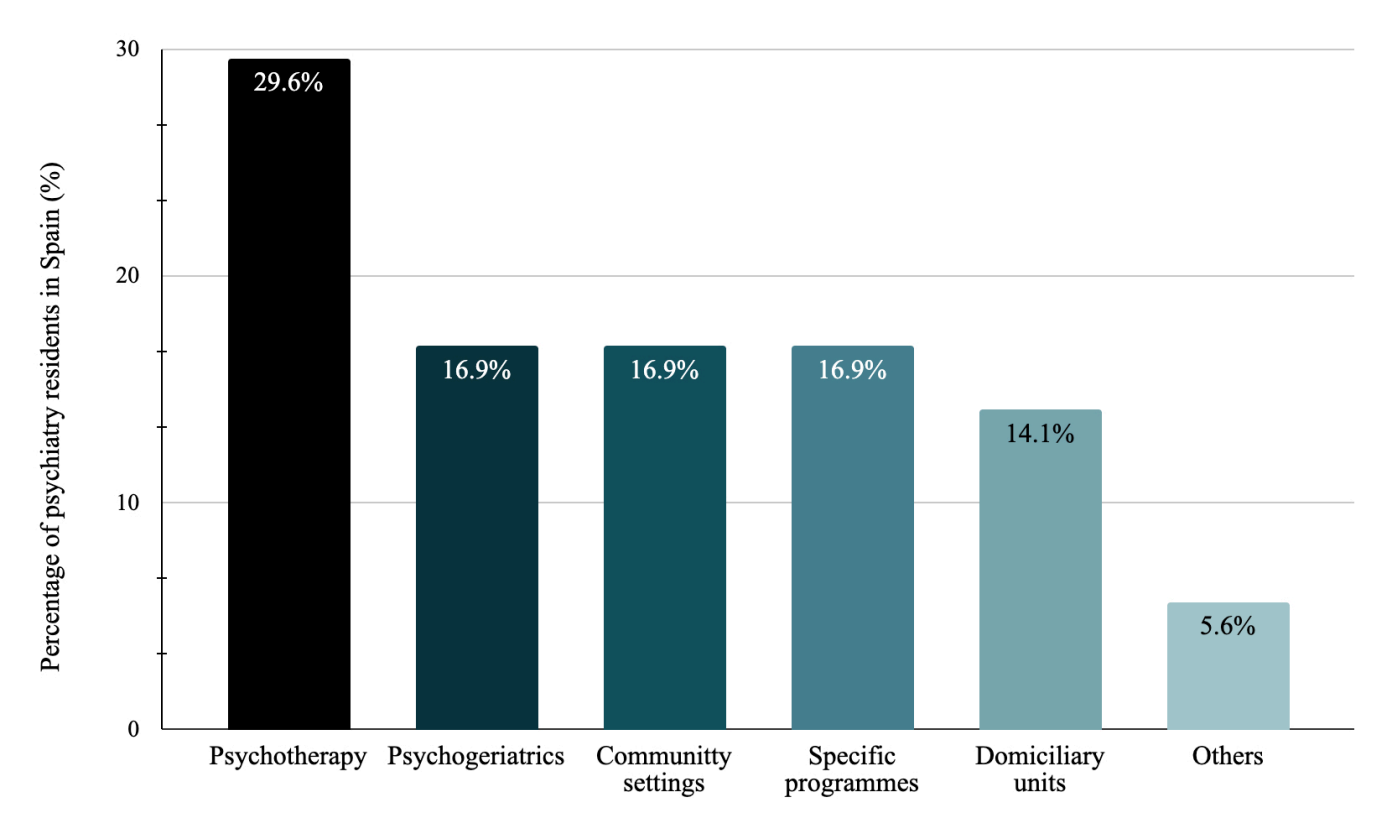
**Percentage of psychiatry residents in Spain who considered that specific subjects should be included in the programme as mandatory rotations**.

The duration of rotations established by the national programme was reported as unfulfilled in 38.5% of training units. Regarding which rotations should have an increased duration, child psychiatry (44.0%) and addictions (52.3%) were the most frequently reported (Fig. 2). In terms of new rotations that should be included as mandatory in the training programme, psychotherapy (29.6%) and psychogeriatrics (16.9%) were the most demanded (Fig. 3).

### Supervision

**Fig. 4.**
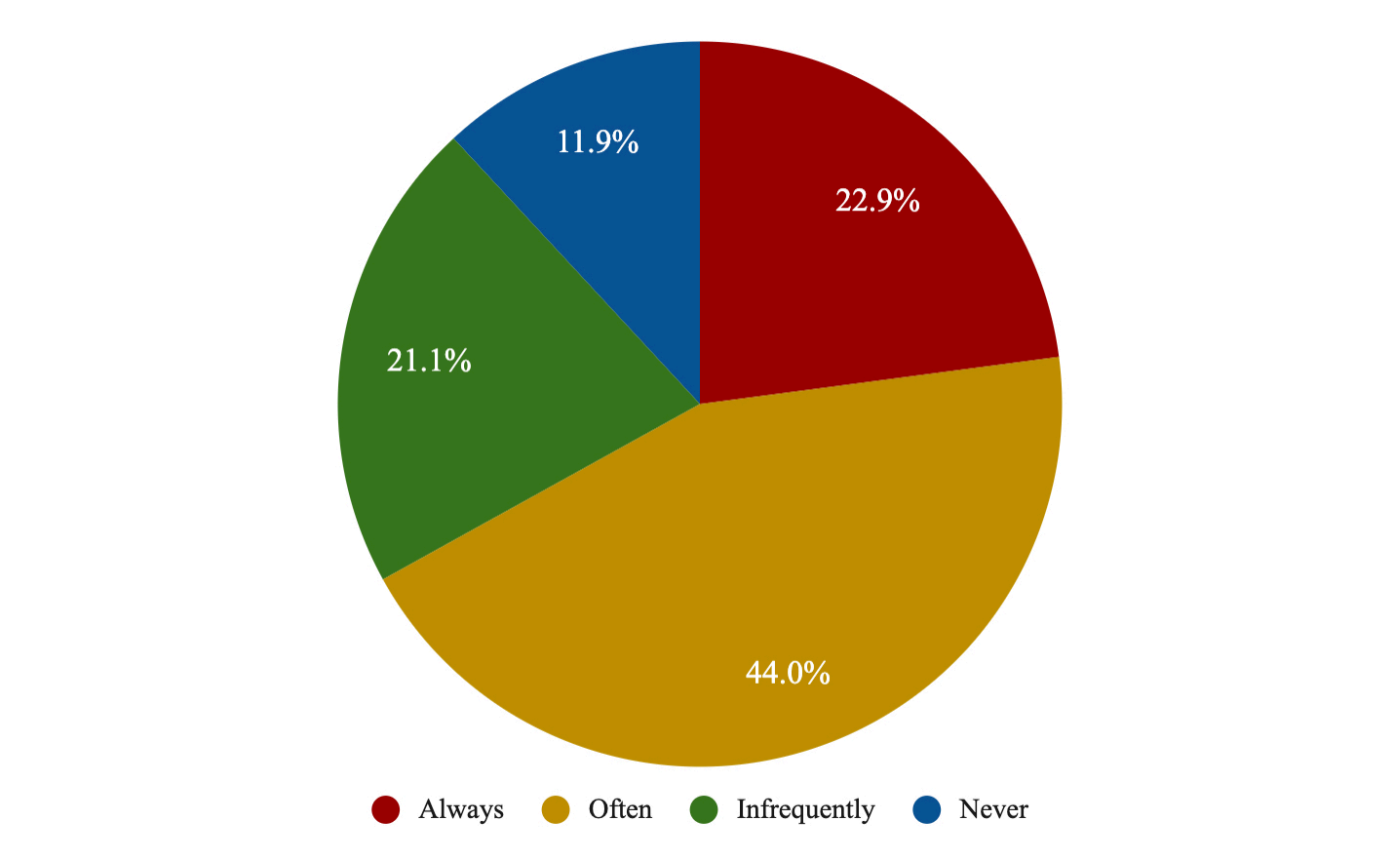
**The perceived frequency of supervision during the first residency year among psychiatry residents in Spain**.

**Fig. 5.**
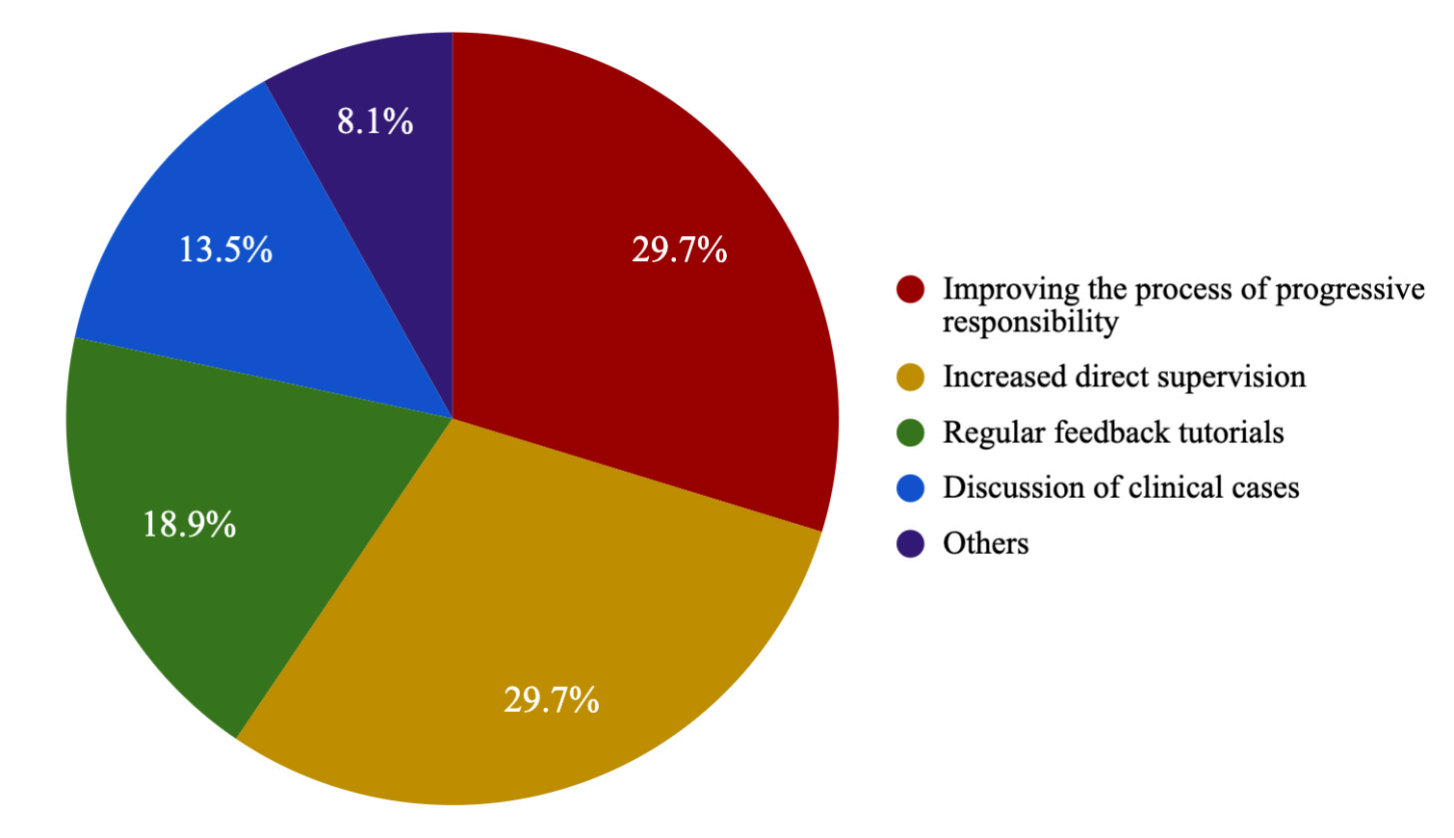
**Percentage of psychiatry residents in Spain who proposed each idea for improving supervision and autonomy**.

Only 22.9% of psychiatry residents reported that they had always been supervised in person during their PGY1; 11.9% said that they had never been supervised during their first year, and 21.1% said that they had been supervised only infrequently; 44.0% had been supervised frequently (Fig. 4). Among students in PGY2–4, the mean score for perceived adequacy of supervision and autonomy was 6.6 ± 2.4. As for proposals to improve supervision, 29.7% of residents proposed the need to enhance the process of responsibility and progressive autonomy, along with an increase in the duration of direct supervision (29.7%) and regular feedback tutorials (18.9%) (see Fig. 5).

### Working Conditions

**Fig. 6.**
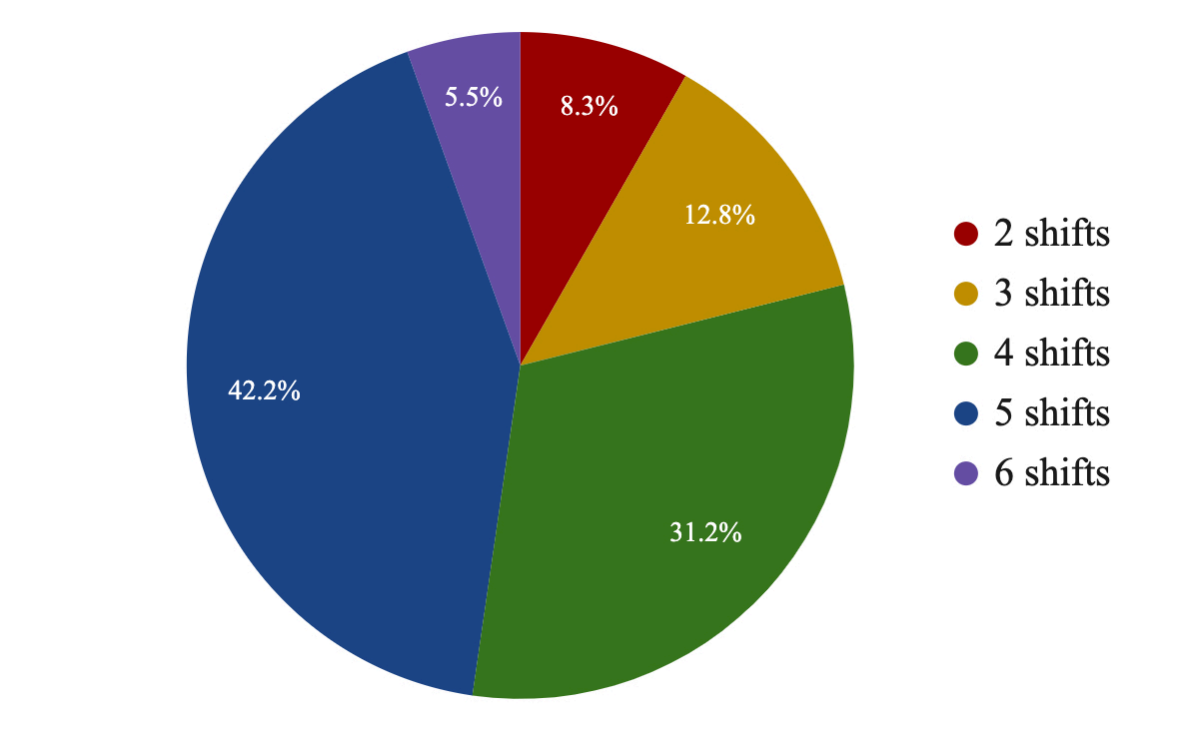
**Percentage of psychiatry residents in Spain who worked different numbers of shifts per month**.

Residents reported an average of 4.3 ± 1.1 (mean ± SE) shifts per month. Concerning the distribution, the most common was five shifts per month (42.2% of residents), followed by four (31.2%), three (12.8%), and two shifts per month (8.3%, see Fig. 6). A smaller number (5.5%) reported working six shifts per month. These data imply that 47.7% of residents exceed four shifts per month, which corresponds theoretically to the maximum established by the European Working Time Directive of 48 working hours per week, according to the latest study on this topic [33]. Regarding the mandatory daily post-shift rest period, 13.8% of residents did not always observe the mandatory daily rest after a shift. In this context, the majority of residents (45.0%) considered that the optimal number of shifts is three; four shifts per month was the second most voted option (30.3%).

## Discussion

To our knowledge, this study is the first in many years to analyse the overall satisfaction and opinions of psychiatry residents regarding their training programme, whether in Spain or elsewhere. In general terms, the satisfaction level of psychiatry residents in Spain regarding competence acquisition seems to be acceptable (score of 6.9 ± 2.4), although satisfaction regarding transversal objectives was low (5.4 ± 2.6), and a large proportion of residents (45.6%) considered that their psychotherapy training was deficient.

The results of this study generally align with the international literature on opinions among psychiatry residents about their training. This alignment may indicate the existence of common, widespread training deficits; the universality or particularity of these educational deficits could be an area for future research. In the case of Spain, trainees seem to be more dissatisfied with the (lack of) psychotherapy training than in other studies [14, 15, 16, 17, 18]; the same applies to clinical supervision [9, 10], particularly regarding the widespread absence of direct supervision during the first residency year. Perceived deficits in research or ethics training were also evident, albeit less so than in previous studies [18, 22]. Other specific areas that did not emerge prominently in this study when compared to the international literature were electroconvulsive therapy [20], psychiatric emergencies [25], psychosomatic psychiatry [26], or care for transgender individuals [27]; this may be because residents did not consider improvements in these areas as a priority when they were asked about their training in general, but when specifically addressed, they may constitute areas for improvement.

Our survey was conducted shortly before the approval of the new training programmes for adult psychiatry and child and adolescent psychiatry in Spain. Therefore, this research helps to understand how psychiatry residents evaluate their training at the end of the previous programme’s life cycle [6]. That programme has been in force for fifteen years (2008–2023, albeit still partially in use until 2026) [8]. The most recent survey, which was published in 2011 [30] but carried out three years earlier, in the final period of the previous programme (1996–2008) in Spain, can serve to assess changes or similarities in terms of residents’ opinions. In this regard, many current criticisms happen to be remarkably similar. The already-expired 2008 programme [6] was proposed (at the time) as an instrument to improve training and satisfy residents’ requests (such as training in psychotherapy), with uneven results and a good part of those pending challenges (such as, again, training in psychotherapy) persisting. In the same way, other areas that were highlighted more than a decade ago as having significant room for improvement—psychotherapy, research methodology, and geropsychiatry—were repeated in the current study [30]. Moreover, when compared to previous articles on medical residents’ working conditions in Spain [33, 34], the results of this study are once again very much in line: as in other medical specialities, psychiatry residents report that institutions often fail to comply with regulations regarding maximum working hours and minimum rest periods.

Our results also reveal a worrying trend regarding supervision: overall, it was rated better in 2008 (mean score = 7.1) than it was in our study (6.5). It is worth mentioning that multiple authors have regarded direct supervision as one of the primary needs for proper psychiatry training [41]; moreover, national regulations establish that direct supervision is always mandatory during the first residency year. Thus, the reported real-life lack of direct supervision during the first residency year is particularly troubling: less than a quarter of the respondents reported that they had received full direct supervision during that period, while a further third reported that they had received it infrequently (21.1%) or even never (11.9%). However, we must consider not only the much-needed improvement in the amount of time dedicated to direct supervision but also optimising its quality [42]. This endeavour must include interventions aimed at improving formative feedback, which is one of the great drivers of learning.

The apparent stalemate (if not worsening) of training provision and working conditions raises critical questions about its causes [43]. It is usually challenging to modify training and organizational dynamics, as resistance to change is commonplace, but the absence of an external control system to audit and evaluate the quality of training in the Spanish medical residency system could be playing a significant role in this lack of progress [44].

The recently approved national psychiatry programme may be an opportunity to improve training and respond, at least in part, to the demands of residents [45, 46]. Indeed, rotations on day hospital and geropsychiatry (two of the most demanded rotations) have been established as mandatory [46], whereas the already mandatory child and adolescent psychiatry rotation (the second most demanded) has seen an increase in training duration under the new adult psychiatry programme, and not only in the new speciality of child and adolescent psychiatry [45]. This programme also establishes a number of 3–4 mandatory shifts per month, in tune with the trainee’s preferences [34]. In any case, we should bear in mind that an element being made mandatory in the national programme does not necessarily imply that it is implemented in actual training, as our results demonstrate.

On the other hand, psychotherapy has not been included in the new training programme as a mandatory rotation. It has been omitted despite it being regarded by residents as the most deficient area and, thus, the rotation that residents deemed most necessary. However, numerous articles have described successful educational interventions in psychotherapy training that could be used in Spanish training units to improve this situation [47, 48]. The duration of rotations in addiction and dual pathology has remained unchanged despite the trainees’ perception that they should be increased. Regardless of the programme clauses, it would be helpful to report successful interventions and best practices that can be used among other training units to enrich the learning process of psychiatry residents elsewhere [49].

All the comments made above should be contextualised within the limitations of our study. First, the fact that participation was voluntary means that there may have been a self-selection bias: the residents who were most dissatisfied with their training might be the most likely to respond to a survey on this topic. However, we suggest that the risk of the sample not being representative is minimised by both its large size (10% of the total population of psychiatry residents in Spain) and its diversity (50% of all the training units across the country). Second, the questionnaire used was the result of an ad hoc development for this project, for which no statistical test has been conducted to assess the reliability or validity of the results; therefore, comparisons with other studies that used other questionnaires must be made with caution. Nonetheless, it should be remembered that the heterogeneity of the questionnaires is the norm in this type of work due to the diversity of objectives or aspects of interest considered by each research group according to their national context.

Regarding its specific design, the survey mainly consisted of general questions and a few specific questions; this means that there may be training areas that residents did not consider but that nevertheless may turn out to be deficient or require further development. However, the study aimed to capture the subjective perceptions of current psychiatry residents, not to provide a comprehensive, multifaceted review of the entire Spanish psychiatry training system; such a task would have been far beyond the scope of this survey. Finally, given the large number of training units whose residents participated in the study, the sample presents remarkable heterogeneity, as shown in Table 2. Because of this, some training units may not be fully represented by the results presented. However, since the study’s objective is to provide an overall picture of the opinions of psychiatry residents in Spain, and as responses have been obtained from a substantial number of training units and residents, the study’s objective is largely reflected in the results. It would be of great interest to conduct specific follow-up studies on those training units whose results differ from the majority.

## Conclusions

In conclusion, psychiatry residents in Spain seemed to be reasonably satisfied with their training, although they identified a series of deficits. There continues to be a series of unsolved, pending challenges in psychotherapy training despite critical shortcomings being identified more than a decade ago by residents and the national committee alike. Additionally, a significant percentage of residents report that regulations regarding clinical supervision continue to be neglected, as do work and rest times. The extent to which the new programme will or will not serve to correct these and other issues remains to be determined. In any case, a window of opportunity is now open to delve into necessary improvements in the training of psychiatry residents in Spain.

## Availability of Data and Materials

The database can be obtained upon reasonable request from the corresponding author.
